# A Review of the Application of Natural and Synthetic Scaffolds in Bone Regeneration

**DOI:** 10.3390/jfb14050286

**Published:** 2023-05-20

**Authors:** Sok Kuan Wong, Michelle Min Fang Yee, Kok-Yong Chin, Soelaiman Ima-Nirwana

**Affiliations:** Department of Pharmacology, Faculty of Medicine, Universiti Kebangsaan Malaysia, Jalan Yaacob Latif, Bandar Tun Razak, Cheras, Kuala Lumpur 56000, Malaysia; michelleyeeminfang@gmail.com (M.M.F.Y.); chinkokyong@ppukm.ukm.edu.my (K.-Y.C.); imasoel@ppukm.ukm.edu.my (S.I.-N.)

**Keywords:** bioglass, bovine bone, calcium phosphate cement, chitosan, hydrogel, hydroxyapatite, polymers, polymethyl methacrylate

## Abstract

The management of bone defects is complicated by the presence of clinical conditions, such as critical-sized defects created by high-energy trauma, tumour resection, infection, and skeletal abnormalities, whereby the bone regeneration capacity is compromised. A bone scaffold is a three-dimensional structure matrix serving as a template to be implanted into the defects to promote vascularisation, growth factor recruitment, osteogenesis, osteoconduction, and mechanical support. This review aims to summarise the types and applications of natural and synthetic scaffolds currently adopted in bone tissue engineering. The merits and caveats of natural and synthetic scaffolds will be discussed. A naturally derived bone scaffold offers a microenvironment closer to in vivo conditions after decellularisation and demineralisation, exhibiting excellent bioactivity, biocompatibility, and osteogenic properties. Meanwhile, an artificially produced bone scaffold allows for scalability and consistency with minimal risk of disease transmission. The combination of different materials to form scaffolds, along with bone cell seeding, biochemical cue incorporation, and bioactive molecule functionalisation, can provide additional or improved scaffold properties, allowing for a faster bone repair rate in bone injuries. This is the direction for future research in the field of bone growth and repair.

## 1. Introduction

Healthy bone undergoes the proper coupling of bone resorption with osteoclasts and bone formation with osteoblasts to achieve physiological bone remodelling and systemic mineral homeostasis [1]. It also possesses a strong regeneration ability against bone injuries or deformities. Delayed healing occurs as a result of incompetent bone regeneration in the presence of osteoporosis, bone tumour removal, large segmental bone defect, infection, and disrupted vascularisation [2]. Hence, advancements in orthopaedic technologies and implanted biomaterials with superior properties is vital to support the mechanical stress experienced and facilitate bone defect healing.

A bone scaffold serves as a temporary platform that provides structural support, facilitates bone repair, and guides bone growth in bone defects [3]. To investigate the osteogenic properties of a bone scaffold, it is often used by researchers as a three-dimensional experimental model cultured with bone cells that better represents the in vivo skeletal microenvironment. Biocompatibility, biodegradability, biomineralisation, osteoinductivity, osteoconductivity, osteogenesis, osteointegration, porosity, interconnectivity, stability, mechanical characteristics, controlled swelling, sterilability, and deliverability of bioactive molecules/drugs are the considerations for the development of bone scaffolds [4,5]. With these features, a scaffold allows bone cell migration, adhesion, invasion, proliferation, and osteogenic differentiation, as well as creating a structure with the mechanical strength and optimum environment for the release of growth factors, the dissemination of nutrients and oxygen, and the coordinated regeneration of bone tissues.

Designing a scaffold which facilitates the structural and functional restoration of the bone remains a critical challenge. Scaffolds are usually derived from living organisms or from synthetic origins. A natural scaffold has similar extracellular matrix characteristics to the bone, superior biocompatibility and bioactivity; however, the variability, batch-to-batch inconsistency, and ethical concerns raised by using animal-derived scaffolds make them less desirable. On the other hand, a synthetic scaffold enables the modification, consistency, reproducibility, availability, and selection of low-cost materials. Previous reviews have provided detailed descriptions of the characterisation of natural and synthetic polymer-based nanocomposites, as well as scaffold design and fabrication [6,7]. The current review aims to describe the use of natural and synthetic scaffolds in bone tissue engineering. The advantages, disadvantages, challenges, and future research direction of the application of bone scaffolds in this rapidly advancing field are also discussed. We hope to provide the readers with a comprehensive overview of the selection of suitable bone scaffolds for research purposes and clinical applications.

## 2. The Application of Natural Scaffolds

### 2.1. Animal and Human-Derived Scaffolds

Scaffolds originating from animals (such as bovine, cuttlefish, fish, and cockle) and humans have been used by researchers to examine their effects on bone regeneration (Table 1).

The trabecular region of animal and human bone can be used to develop bone scaffolds. However, bones derived from one host induce an immune response after implantation in another host [8]. For instance, cellular components are considered foreign antigens across species that lead to the expression of major histocompatibility complexes, leading to graft rejection [9]. Decellularisation is the process of removing cellular components from the native bone matrix while preserving its microarchitecture and composition [10]. Decellularised native bone does not trigger an immune response in the host as compared to unprocessed native bone [10]. The well-preserved extracellular matrix in the decellularised native bone provides structural and mechanical properties to the tissue as well as supporting cell growth and survival of the functional tissue or organ [11]. The common approaches used for bone decellularization include chemical, biological, and/or physical treatments. The agents used for chemical treatment include cell-lysing non-ionic or ionic detergents, such as sodium dodecyl sulphate, ethylenediaminetetraacetic acid, and Triton X-100 [12]. The agents used for biological treatments include nucleases, such as deoxyribonuclease or ribonuclease, for enzyme digestion [10]. On the other hand, physical treatment involves freezing, direct pressure, sonication, agitation, and vacuum-assisted decellularisation. Physical treatments destroy the cell membrane while reducing extracellular matrix damage and ultrastructure disruption [13].

Several groups of investigators have developed decellularised bovine bone scaffolds using a combination of different decellularization methods. Shahabipour et al. obtained cancellous bone scaffolds (5 mm diameter × 2 mm height) from the epiphyseal of the bovine femur, which was subjected to immersion in hot water for lipid removal and freeze–thaw cycles for cell lysis. After that, chemical treatment ensued using sodium dodecyl sulphate with gentle agitation. The complete removal of cells with similar structural properties to native bones were noted after the decellularisation process. Histological analysis and scanning electron microscopy showed increased cell growth, adhesion, and integration on the decellularised bone scaffold after seeding with rat bone marrow mesenchymal stem cells [14]. Bianco et al. employed a blend of physical and biological processes to produce decellularised bone marrow scaffolds from bovine metatarsal and metacarpal bone. The scaffolds displayed well-preserved adipose tissues, vessels, collagen fibres, and the absence of cell fragments after being subjected to freeze–thaw cycles and enzymatic digestion. A comparable mechanical strength was detected in both the native and decellularised tissues. An increased expression of stromal cell-derived factor 1, stem cell factor, and hepatocyte growth factor were also observed, facilitating the proliferation and adhesion of human bone marrow stromal cells (hBMSCs) onto the bone scaffold [15].

Bone demineralisation is another strategy used along with bone decellularisation to improve the preparation of bone scaffolds from natural sources. It is a process of removing fat residues, impurities, and inorganic minerals while leaving the collagen matrix and bone morphogenetic proteins in the bone [16]. Demineralised bone scaffolds possesses more potent osteoinductive properties than non-demineralised bone scaffolds as the demineralisation process enhances the bioavailability of osteogenic proteins, thus inducing cell differentiation and encouraging new bone formation [17]. The common approaches used for bone demineralisation include chemical treatments using alcohol to remove excess fat from the bone and hydrochloric acid to remove calcium content from the bone [18].

A study by Shi et al. demonstrated that the demineralised bone matrix derived from bovine bone had good degradation activity as well as providing a suitable microenvironment for cell attachment and bone mineralisation after 14 days of incubation with human umbilical cord mesenchymal stem cells [19]. Recently, Jolly et al. created a three-dimensional bone scaffold obtained from bovine trabecular bone, followed by co-culturing with human foetal osteoblasts (hFOB1.19) and human peripheral blood mononuclear cells (hPBMCs). Their findings proved that decalcified bovine bone scaffolds with osteoblast–osteoclast co-culture supported osteoblast cells proliferation, improved bone mineral density (BMD), and bone mineral content (BMC) [20].

Apart from bovine bone, cuttlefish is a marine vertebrate and the bone of cuttlefish can be used to develop bone scaffold. Cuttlefish bone consists of calcium carbonate mimicking the chemical, crystallographic structures, and morphology of the mineral phase of natural bone [21]. Cuttlefish is lower in cost and abundantly available. Battistella et al. fabricated hydroxyapatite porous scaffolds via the hydrothermal transformation of cuttlefish bone from *Sepia officinalis*. The hydrothermal transformation approach converts the calcium carbonate in cuttlefish bone into calcium phosphate (hydroxyapatite). The scaffold was tested with mouse osteoblast-like cells (MC3T3-E1) for the expression of osteogenic markers. The level of alkaline phosphatase (ALP) was increased at an early stage of cell differentiation and the expression of osteocalcin (OCN) was elevated at the later stage of osteogenesis. These results reiterated that the hydroxyapatite porous scaffolds derived from cuttlefish bone support the expression of bone-related proteins involved in osteoblast cell proliferation and bone calcification [22].

Fish scales are biowaste materials that can be processed into hydroxyapatite powder. A scaffold made from fish scales mimics the crystallographic structure, phase, composition, and morphology of native bone [23]. A natural hydroxyapatite scaffold was prepared by Mondal et al. using fish scales from tilapia fish (*Oreochromis mossambicus*). The scaffold was characterised by its mechanical behaviours and biological properties. The findings showed that the fabricated scaffold exhibited higher tensile and compressive strength compared to the native bones from humans, cattle, swines and horses. An in vitro experiment culturing human osteoblast cells (MG63) on the fish scales-derived hydroxyapatite scaffold found similar cell viability to the control hydroxyapatite without fish scales. The scaffold was tested in vivo by implanting the test materials into the bone defect created at the cortex region of the rabbit’s left femur. The microscopic view of the bone defect region showed good bio-affinity and osteoconductivity, evidenced by the infiltration of cells into the materials and the presence of new cell lining [24].

Cockle shells are biowaste products from consumed cockles or molluscs, such as mussels or scallops. Cockle shells consist of the pure aragonite form of calcium carbonate polymorph that can be replaced by bones, making it suitable to be used as bone scaffolds [25]. A study carried out by Bharatham et al. used cockle shell powder incorporated with alginate to develop nanobiocomposite bone scaffolds. The scaffold, consisting of 40% alginate and 60% nano cockle shell powder, demonstrated favourable characteristics as bone graft substitutes with adequate pore size for cell migration and vascularisation, moderate degradation rate, and high mechanical strength. Cell proliferation of MG63 was promoted when seeded on the scaffold [26].

A study by Mebarki et al. investigated the characteristics of hBMSCs when cultured onto Tutoplast^®^-processed human bone. It is a bone graft generated through an alkaline treatment for delipidisation, an osmotic treatment to break down the cell wall, an oxidative treatment to remove the immunogenic structures of viruses, solvent dehydration to preserve the natural tissue matrix, and a γ-irradiation procedure to sterilise the graft. Cells seeded onto the Tutoplast^®^-processed human bone showed good cell adhesion and higher osteoblastic gene expression after a week. The subcutaneous grafting of biomaterial containing hBMSCs in 7-week-old severe combined immunodeficient (SCID) mice resulted in bone neoformation as early as two weeks after implantation [27].

Researchers have attempted to combine several natural materials to establish new bone scaffolds. Collagen is the most abundant protein in the human body and a main component of the connective tissues, including tendons, ligaments, and bones. It appears as an ideal material for bone repair mainly attributable to its compatibility, degradability, strong plasticity, and low immunogenicity. The combination of collagen with other bioactive materials is often preferable because pure collagen lacks geometrical properties due to its low biomechanical strength and exerts insufficient osteogenic activity to stimulate bone formation [28], hence limiting its clinical application as a bone graft by itself. Natural polymers, such as glycosaminoglycans, are usually combined with collagen to form composite scaffolds. A collagen–glycosaminoglycan scaffold synthesised via the mixing of collagen type I (COL1) from bovine tendon and chondroitin-6-sulphate from shark increased bone formation in adult male Wistar rats with a 15 mm calvarial defect. Comparable defect healing rates and areas of new bone formation were also seen in the animals implanted with collagen–glycosaminoglycan scaffold as compared to autogenous bone [29]. In cell cultures, the collagen–glycosaminoglycan scaffold allowed cell growth and infiltration when seeded with MC3T3-E1 cells [30].

Recently, a biomimetic composite scaffold was developed using an eggshell membrane as a template with the addition of tricalcium phosphate from bovine bone ash, gelatin, and chitosan [31]. Gelatin is a biodegradable and biocompatible polymer that promotes the adherence of cells with minimal antigenicity. Chitosan is a linear polysaccharide derived from natural chitin abundantly found in the exoskeletons of insects, algae, and fungi’s cell wall [32]. Chitosan is used as a scaffold by researchers because it mimics the extracellular matrix of mineralised tissue. Chitosan is highly biocompatible and non-toxic, as well as capable of providing excellent cell adhesion, cell proliferation, and new bone tissue formation. Gelatin and chitosan function as an organic matrix where the composite material stimulates a cellular response by accelerating the bone healing process. In the study conducted by Neacsu et al., the cellular viability and oxidative stress status in the culture containing amniotic fluid stem cells and the scaffold were determined. The investigators reported a higher viability for the amniotic fluid stem cells seeded on the composite scaffold than those without scaffolds. The glutathione synthesis was also not inhibited, indicating the lower oxidative stress level of the amniotic fluid stem cells in the presence of the composite scaffold [31].

In summary, natural scaffolds originating from animals support cell growth, attachment, proliferation, and functionality. However, the shortcomings of the previous investigations and the use of bone scaffolds from animals need to be acknowledged. Firstly, a static culturing environment is often used on animal bone scaffolds. The lack of sufficient oxygen and nutrient supply and the accumulation of metabolic waste products limit cell growth and form irregular cell density within the scaffold. This problem can be solved by developing a bioreactor system which provides a dynamic environment for cell-loaded scaffold culture [33]. Secondly, its low mechanical properties result in the limited application of decellularised animal bone scaffold. As a solution, synthetic polymers or biological substances have been incorporated to improve the features of the animal bone scaffolds. Thirdly, animal bone scaffolds obtained from different animal sources and different parts of interest may differ in structure and mechanical stability. This inconsistency results in the difficulty of predicting the microstructural improvement of the studied bone scaffold. Thus, the standardisation of bone scaffold is recommended. Scaffold architectural features such as shape, dimension, porosity, and geometry should be taken into consideration.

**Table 1:** The use of animal-derived scaffold in vivo and in vitro for bone regeneration.

Type of Scaffold	Type of Model	Findings	Reference
Decellularised bone scaffold from the cancellous bovine femur	Rat bone marrow mesenchymal stem cells	-The decellularised scaffolds had no host cells with a bone trabecular-to-bone surface ratio similar to the native bone samples. -Cells were distributed between bone trabeculae and cell numbers were increased in the decellularised scaffolds after seeding with rat bone marrow mesenchymal stem cells.	[14]
Decellularised bone marrow scaffold from the bovine metatarsal and metacarpal bone	hBMSCs	-The decellularised scaffold had no cells and well-arranged bone marrow extracellular matrix components, including adipose tissues, vessels, collagen III, collagen IV, and fibronectin. -The mechanical strength of the decellularised scaffolds was of the same magnitude as that of the native bones. -The decellularised scaffold supported cell growth and adhesion.	[15]
Demineralised bovine bone matrix scaffold	Human umbilical cord mesenchymal stem cells	-The demineralised scaffold had a lower remaining weight after 14 days of incubation with the cells. -The demineralised scaffold supported cell attachment and biomineralization.	[19]
Demineralised bovine trabecular bone scaffold	Co-culture of hFOB1.19 and hPBMCs	-Successful colonisation of osteoblasts on the demineralised scaffold. -The demineralised scaffold had increased BMD and BMC.	[20]
Cuttlefish bone scaffold	MC3T3-E1 cells	-The expressions of ALP and OCN were increased.	[22]
Fish scale-derived hydroxyapatite scaffold	MG63 cells	-The cells cultured on the fish scale-derived hydroxyapatite scaffold and the control hydroxyapatite without fish scales had similar viability.	[24]
	Albino rabbit with three bone defects (2 mm each) at the cortex region of the femur	-Infiltration of new cells onto the scaffold and new cell lining were noted at the defect site implanted with fish scale-derived hydroxyapatite scaffold.	
Cockle shell powder nanobiocomposite bone scaffold	MG63 cells	-The scaffold had an ideal pore size range (50–336 μm), a moderate degradation rate, increased compressive strength, elasticity, and cell proliferation rate.	[26]
Tutoplast^®^-processed human bone	Male SCID mice (*n* = 6, 7 weeks old)	-Increase in bone formation and cell adhesion on the Tutoplast^®^-processed human bone than on the hydroxyapatite/β-tricalcium phosphate (β-TCP).	[27]
COL1 + chondroitin-6-sulphate	MC3T3-E1 cells	-Allowed cell growth and cell infiltration.	[30]
	Male Wistar rats with calvarial defect	-A higher bone area and percentage of bone healing were seen in the defects grafted with collagen–glycosaminoglycan scaffold than in the defects that were left empty. -A similar bone area and percentage of bone healing were seen between the groups implanted with collagen–glycosaminoglycan and autologous bone.	[29]
Composite scaffold (eggshell membrane + bovine bone ash + gelatin + chitosan)	Amniotic fluid stem cells	-A higher cell viability and lower oxidative stress level in the cells cultured on the composite scaffold.	[31]

Abbreviations: β-TCP, β-tricalcium phosphate; ALP, alkaline phosphatase; BMC, bone mineral content; BMD, bone mineral density; hFOB1.19, human foetal osteoblasts; hPBMCs, human peripheral blood mononuclear cells; MC3T3-E1, mouse osteoblast-like cells; MG63, human osteoblast cells; OCN, osteocalcin; SCID, severe combined immunodeficient.

### 2.2. Plant-Derived Scaffolds

Bone scaffolds from plant tissue serve as an alternative to scaffolds obtained from animals and humans, overcoming the issues of immunogenicity and ethical concerns about the use of animal- and human-derived bone scaffolds.

Various three-dimensional cellulose constructs of plants (such as apple, broccoli, sweet pepper, carrot, persimmon, and jujube) with various sizes and shapes have been decellularised and tested for their ability to serve as scaffolds to enhance osteoblast growth and proliferation (Table 2). Among these scaffolds, apple has been proven useful in culturing pluripotent stem cells with increased cell numbers and proliferation as compared to other plant-derived scaffolds. The pluripotent stem cells were then cultivated in an apple-derived scaffold supplemented with osteogenic differentiation media. The findings showed increased mineralising nodules and expression of osteogenic markers. In a rat calvarial-defect model grafted with an apple-derived scaffold, bone regeneration was visualised with the presence of collagen deposition and blood vessel formation at the implanted area [34]. Contessi Negrini et al. produced a vegetable-derived scaffold using carrots for bone tissue engineering. Carrot scaffolds were obtained from the xylem of the transversal section. The scaffolds were treated with sodium dodecyl sulphate and went through sonication for 5 min at 40 °C [35]. In vitro direct cytocompatibility tests were conducted using pre-osteoblast MC3T3-E1 cells. The cells were cultured in an osteogenic medium to induce osteogenic differentiation after cell seeding. Viable cell growth was seen on the decellularized carrot scaffold. Furthermore, an increase in ALP was observed in the cells cultured in an osteogenic medium on decellularised carrots [36].

**Figure 1:**
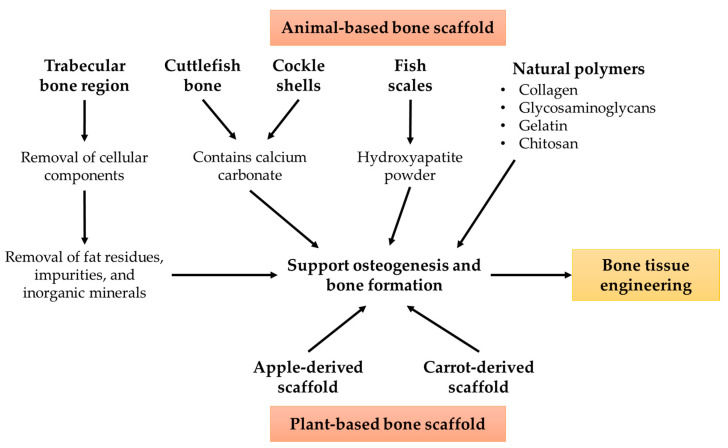
The properties of natural scaffolds in bone tissue engineering.

Plant materials used for scaffold establishment can be obtained conveniently in a mass as they are abundantly grown in various topographies on earth. Similar to animal-derived scaffolds, plant-derived scaffolds have been proven to have bone regeneration properties. However, their mechanical properties may be considerably lower than those of natural bone tissue. Plant scaffolds could be applied clinically as bone filler in non-load-bearing sites. In addition, their combination with other inorganic biocomposites (such as hydroxyapaptite) could be considered to enhance their mechanical strength. The properties of natural scaffolds in bone tissue engineering have been summarised in Figure 1.

## 3. The Application of Semi-Synthetic and Synthetic Scaffolds

### 3.1. Calcium Phosphate Cement and Hydroxyapatite

Calcium phosphate cement is a bioactive grafting material typically used in orthopaedic applications for bone regeneration due to its similarity to natural bone mineral composition. It exists in the form of a powder and sets as hydroxyapatite upon the addition of a liquid phase. Calcium phosphate cement and hydroxyapatite exhibit great biocompatibility, osteoconductivity, resorbability, self-setting ability, injectability, and mouldability. It can act as a controlled drug carrier [37]. Many studies have tested the use of calcium phosphate cement and hydroxyapatite in bone regeneration in vitro and in vivo.

Chen et al. studied the efficacy of β-TCP scaffold co-cultured with human bone marrow stem cells and periosteal-derived stem cells in athymic mice. The insertion of a scaffold with co-cultured cells into the subcutaneous dorsal surface of CD-1^®^ nude mice resulted in increases in total bone formation, mature bone formation, and neovascularisation [38]. A study by Mebarki et al. used a hydroxyapatite/β-TCP scaffold seeded with hBMSCs followed by implantation into the subcutaneous pockets of SCID mice. The results showed that newly formed bone was detected and the expression of osteogenic genes was upregulated as early as two weeks after grafting [27].

Leventis et al. created a circular bicortical critical-size cranial defect in male New Zealand white rabbits, which was grafted with a synthetic scaffold containing β-TCP and calcium sulphate. Six weeks after implantation, the grafted material was observed to have incorporated with the bone and showed no inflammatory responses. Histological results showed no decrease in the osteoblast number (Ob.N), osteoblast perimeter (Ob.Pm), or fibrous connective tissue area (Fb.Ar), but showed a decrease in the graft volume/tissue volume (Gr.V/TV) and graft area (Gr.Ar), and marginal increases in BV/TV, osteoid volume/tissue volume (OV/TV), bone area (B.Ar), osteoid area (O.Ar), osteoid perimeter (O.Pm), osteoclast number (Oc.N), and mineralisation. The biphasic scaffolds composed of β-TCP and calcium sulphate showed excellent biocompatibility, osteoconductivity, and biodegradability [39].

The clinical application of calcium phosphate cement and hydroxyapatite has also been reported. An earlier study carried out by Mattson et al. indicated that the augmentation of calcium phosphate cement improved the stability of fractures by lowering fracture movement and rotation in patients with unstable trochanteric fractures (aged 66–95 years old) [40]. Recently, a study by Kim et al. reported that the surgical procedure of volar locking plate fixation with an injection of calcium phosphate bone cement in patients with unstable distal radial fractures (aged ≥65 years) enabled all the fractures to heal uneventfully, and did not cause tendon-related complications or non-unions postoperatively [41].

The major limitations of calcium phosphate cement and hydroxyapatite include low mechanical performance, poor washout resistance, and lack of osteogenic activities. The incorporation of other materials into calcium phosphate cement and hydroxyapatite has been proven to overcome these shortcomings and/or enhance the original features of the biomaterials [37,42]. Nair et al. developed a composite scaffold comprising hydroxyapatite, tricalcium phosphate, and calcium silicate. The purpose of adding silica to the composite in their study was to improve the resorption ability, as the silica-based bioceramic is a better biodegradable material than hydroxyapatite alone. The seeding of goat bone marrow-derived mesenchymal stem cells showed higher cell viability, adhesion, and proliferation in the scaffolds with calcium silicate addition compared to those without calcium silicate [43].

In addition, Shi et al. established a three-dimensional scaffold consisting of dopamine-modified alginate and quaternised chitosan-templated hydroxyapatite. The scaffold was created to enhance the biomechanical strength of the scaffold. The bone-repairing properties of the scaffold were studied in both in vitro and in vivo studies. The dopamine-modified alginate and quaternised chitosan-templated hydroxyapatite was shown to support cell adhesion and the growth of human chondrocytes and fibroblasts. Using an in vivo model of New Zealand white rabbits with femoral bone defects, the implanted scaffold promoted new bone formation and accelerated defect healing [44].

A recent study by Dulany et al. evaluated the in vitro and in vivo behaviours of β-TCP added with poly(1,8-octanediol-co-citrate) and cerium oxide nanoparticles using primary human osteoblasts and a healthy rat model. Findings from the in vitro study indicated promising cell attachment, low cytotoxicity, increased cell proliferation, and mineralisation of osteoblast cells on nanocomposite scaffolds under normal and oxidative stress conditions. The nanocomposite scaffolds were also implanted subcutaneously into the eight-week-old Sprague–Dawley rats. It was found that the nanocomposite scaffolds exhibited biocompatible properties, evidenced by the presence of cell infiltration throughout the scaffolds and a minimal immune response of the surrounding tissues. In addition, scaffold degradation occurred after 30 days from implantation. The postulated underlying mechanism for bone tissue generation was mediation through the free radical scavenging property, facilitating the reduction of oxidative stress [45].

In conclusion, calcium phosphate cement and hydroxyapatite are commonly used by researchers in scaffold compositions as they provide great biocompatibility to the cells cultured on them. In addition, calcium phosphate cement and hydroxyapatite scaffold were rarely rejected by the animal or human hosts in observations of the union of graft materials with the host bone. Reinforcement utilising biological or synthetic materials also helps in improving the shortcomings and increasing the effectiveness of calcium phosphate cement and hydroxyapatite. The use of calcium phosphate cement and hydroxyapatite scaffold in vivo, in vitro, and in humans is summarised in Table 3.

### 3.2. Bioglasses

Bioglasses, or bioactive glasses, are highly reactive silicone-based glass-ceramic biomaterials that contain calcium and phosphorus. They release ions and form a carbonated hydroxyapatite layer when immersed in biological fluids, promoting protein adsorption for bone formation and implant–bone integration [46]. The bioavailability and bone bonding ability of bioglasses allows them to be directly used as bone grafts for implantation or as an additive to enhance the properties of other bone scaffolds.

An earlier study by Deb and colleagues used bioglass as a graft material in both the mono- and co-culture of primary human osteoblasts and human umbilical vein endothelial cells (HUVECs). The scaffold was fabricated using powdered 45S5 Bio-glass^®^ in polyvinyl alcohol as the porogen. The findings of the in vitro study indicated that bioglass was a good graft material for bone regeneration. There was no cytotoxicity, but increased cell proliferation was reported in the mono- and co-cultures seeded onto the bioglass-derived porous scaffolds. The scaffolds also showed an excellent interconnected porous structure, allowing for excellent cell penetration from the outer to the inner layer of the scaffolds when observed with a scanning electron microscope. The proliferation response of the human osteoblast cells seeded on the bioglass was found to be better than for hydroxyapatite [47]. Gabbai-Armelin et al. fabricated a novel fibrous bioglass scaffold comprising malleable fibres, such as silicone oxide (SiO_2_), sodium oxide (Na_2_O), potassium oxide (K_2_O), magnesium oxide (MgO), calcium oxide (CaO), and phosphorus pentoxide (P_2_O_5_). Their novel biomaterial scaffold was implanted into a non-critical-sized tibial defect of male Wistar rats to investigate the in vivo tissue response. The novel scaffold showed complete degradation over time, a lack of inflammatory response, new bone formation, an increased Runt-related transcription factor 2 (Runx-2) expression, and a higher maximal load [48]. Another study evaluated the bone-healing ability of bioglass scaffolds and powders using male Wistar Lineage rats with a femoral bone defect. As compared to the autogenous bone group, a lower BV/TV and trabecular number (Tb.N), but no change in the trabecular thickness (Tb.Th) or trabecular separation (Tb.Sp), were observed in the bioglass-scaffold group after 30 days from implantation [49].

Given that the bioglass did not achieve comparable bone healing properties to the autografts [49], researchers have attempted to reinforce bioactive glass with other synthetic materials, such as drugs, transition metals, and trace elements. The addition of raloxifene, a selective oestrogen receptor modulator (SERM), into bioglass composite resulted in bone-repairing effects at a rat’s calvaria defect similar to those without raloxifene. These findings showed that raloxifene did not enhance the osteogenic properties of bioglass to a greater extent [50]. Nonetheless, the incorporation of niobium (a transition metal) into bioglass further promoted bone formation at a rat’s femoral defect comparable to that of autogenous bone, evidenced by no significant difference in BV/TV, connectivity density (Conn.D), Tb.Th, or Tb.N between the two experimental groups [49]. Zhang et al. fabricated a boron-containing bioglass scaffold via sol-gel and three-dimensional print techniques. Boron regulates various micronutrients in the body, including calcium, phosphorus, aluminium, and molybdenum, thus suggesting its role in promoting osteogenesis in bone physiology. An in vitro study showed great cell compatibility and proper cell attachment of rat bone marrow mesenchymal stem cells on the fabricated boron-containing bioglass scaffold with uniform-pore size. Furthermore, the in vivo study showed promising results when the boron-containing bioglass scaffold was implanted in a mandibular defect model of male New Zealand white rabbits. The results showed a shorter time was needed for bone healing. The implanted graft was completely degraded with new bone formation. Higher BMD, BV/TV, and Tb.Th were detected at the defect site implanted with the boron-containing bioglass scaffold as compared to the blank control [51].

An in vivo study carried out by Li et al. assessed the effect of baghdadite scaffold unmodified, or modified with polycaprolactone coating containing bioactive glass nanoparticles, on bone regeneration in a sheep model with bone defects. A critical-sized segmented bone defect was created at the right tibia of Merino wethers, and the scaffolds were implanted. The defect site was then subjected to radiographic, biomechanical, micro-computed tomography, and histological assessments after 26 weeks. Both the unmodified and modified baghdadite scaffolds showed similar outcomes for interconnected porosity, bone remodelling, new bone formation within the area of defect, good integration, bone infiltration, and new bone ingrowth. A biomechanical analysis showed no difference in torsional stiffness and ultimate torsional strength between the animals inserted with the unmodified or modified baghdadite scaffolds [52].

In summary, the documented evidence indicates that bioglass is a potential biomaterial for bone tissue regeneration, with an efficacy higher than hydroxyapatite but lower than autologous bone. Bioglass contains alkali metals that provide an optimum alkaline environment which promote osteoblast proliferation and inhibit osteoclast production, thus suggesting it as a biomaterial superior to hydroxyapatite, which is slightly acidic. The clinical uses of bioglass have been extensively implemented in the field of dental and maxillofacial applications [53], with less attention on weight-bearing bones, and thus awaits further validation from human trials. In addition, carbon dots-reinforced hydrogel has been recently developed, and has mechanical robustness, self-healing behaviour, and a better diffusion rate for drug release [54]. Hence, future research to evaluate its bone-regenerative properties are warranted in biological systems. A summary of the use of bioglass as bone scaffolds in vivo and in vitro is presented in Table 4.

### 3.3. Chitosan Composite

Chitosan mimics the glycosaminoglycans of the extracellular matrix which facilitate cellular adhesion. Pure chitosan has weak mechanical strength due to its linear structure in nature. Hence, it is a limiting factor in its use for the purpose of bone tissue engineering [55]. The presence of crosslinkers (such as dextrins, genipins, and purines) is essential to crosslink the fragmented chain of chitosan. Several additives can enhance the mechanical properties of pristine chitosan scaffold while retaining its biocompatibility, biodegradability, mouldability, and anti-bacterial property upon their integration [56].

The combination of commercially purchased chitosan and hydroxyapatite increased the compression strength of the scaffolds as compared to the pure chitosan scaffolds. Seeding with MC3T3-E1 cells resulted in good cell attachment, proliferation, and differentiation on the exterior surface and within the pores of the chitosan–hydroxyapatite scaffold [57]. Similar observations were noted in a study conducted by Jahan et al. They combined chitosan and hydroxyapatite with purine nucleotide as a crosslinker in developing a bone scaffold. The seeding of MC3T3-E1 cells on the chitosan–hydroxyapatite scaffold resulted in increased cell proliferation, ALP activity, osterix expression, and calcium phosphate deposition as compared to the chitosan scaffold only. An in vivo experiment was conducted by implanting a chitosan scaffold with and without the addition of hydroxyapatite at the rod-fixated tibia fracture site in 4-month-old mice. The efficacy of the chitosan–hydroxyapatite scaffold for bone regeneration was superior to the chitosan scaffold, evidenced by denser bone microarchitecture, higher osteoid formation, and the presence of a cartilage matrix [58].

With the continuous advancement of technology, the use of nanoparticles has been a valuable and promising material due to their nanoscale size, high surface area, excellent biocompatibility, and easy functionalisation which fulfils the requirements of bone tissue engineering. Silica serves as a material involved in the early stages of bone calcification by allowing the crystallisation of apatite crystals, cell adhesion, and the formation of collagen. Its nano-sized particles provide a larger surface area which enables interaction with the surrounding bone tissues, hence improving the bioactivity of the composite scaffold [57]. The chitosan–silica hybrid scaffold exhibited robust mechanical strength and non-cytotoxicity towards mice bone marrow stromal cells [59]. Kavya et al. incorporated nano-silica and gelatin into the chitosan scaffold via the lyophilisation process. The maximum tensile strength of the nano-silica/gelatin/chitosan scaffold was increased as compared to the pure chitosan scaffolds. The findings of an in vitro study showed excellent cyto-compatibility of the composite scaffold with MG-63 cells. The nanocomposite scaffold also encouraged better cell attachment and proliferation than the conventional chitosan scaffolds [60].

Nanohybrid technology using several types of nanofillers (such as clay, carbon nanotubes, graphene oxide, and metal ions), has also been adopted to improve the mechanical properties of chitosan. Mahanta et al. fabricated a nanohybrid chitosan scaffold using nanoclay (nanoparticles of layered mineral silicates) and magnesium aluminium phosphate oxide (Mg-Al-PO_4_)-layered double hydroxide (layered materials with divalent or trivalent metal ions). Both nanofillers have opposite surface charges facilitating sustained drug delivery in the chitosan matrix. An in vitro study using mouse embryonic fibroblast (NIH 3T3) cells demonstrated great biocompatibility, mechanical strength, and no cytotoxicity. The rats subjected to femoral bone defects which were filled with the test scaffold showed greater bone healing, denser bone morphology, and higher osteoblast numbers as compared to the rats implanted with pure chitosan scaffolds, with no observable adverse effect on the liver or kidneys [61].

He et al. combined chitosan with polytrimethylene carbonate, polylactic acid, oleic acid-modified hydroxyapatite, and vancomycin hydrochloride to fabricate a microsphere scaffold. The scaffold showed slow biodegradability, extensive osteoblasts adhesion, and enhanced mechanical strength of the scaffold. The presence of oleic acid-modified hydroxyapaptites stimulated the osteogenic proliferation of MC3T3-E1 cells [62].

Generally, low mechanical strength precludes the use of pure chitosan scaffolds for bone repair at load-bearing sites. The modification of chitosan with additives provides better mechanical support, making it a promising material for bone-tissue-engineering applications. A summary of the use of chitosan as a scaffold in vivo and in vitro is presented in Table 5.

### 3.4. Hydrogel

Hydrogel is a three-dimensional hydrophilic polymer network capable of absorbing a large amount of water while retaining its structure. Hydrogel mimics the natural extracellular matrix of indigenous bone that plays multifaceted roles, providing nutrients for endogenous cell growth, conferring structural support at the defective site, entrapping cells and proteins, as well as demonstrating excellent integration with the surrounding tissues to reduce inflammatory responses upon transplantation [63]. Natural biomolecules (such as gelatin, alginate, collagen, hyaluronic acid, and chitosan) are commonly used individually or in combination to prepare hydrogels, which have been reported to be potentially beneficial in bone repair. Although hydrogels contain materials from natural origin, the industrial production of hydrogels requires a great amount of artificial and petrochemical materials (such as acrylic acid and acrylamide) for the simultaneous polymerisation and cross-linking processes [64].

Gelatin is a protein extracted through the hydrolysis of collagen-rich components, exhibiting the feature of brittleness when dry but elastic when moist. Gelatin contains identical cell-binding motifs to collagen, thus serving as an alternative to collagen due to its lower cost [65]. A biomimetic periosteum-bone substitute was established using gelatin methacryloyl hydrogel and its bone-repairing efficacy was tested upon insertion into a large segmental bone defect in New Zealand white rabbits. The animals receiving the hydrogel bone grafts showed higher BV/TV, new bone formation, faster bone healing, regeneration, and remodelling relative to the control group. Furthermore, an in vitro study using bone marrow mesenchymal stem cells showed higher cell proliferation and increased cell chemotactic effect in the hydrogel group. The upregulation of osteogenic genes, such as COL1, ALP, OCN, Runx-2, and OPN, and increased mineral deposition were observed in the hydrogel group compared to the control group [66]. Another study carried out by Fang et al. pointed out that the biomimetic gelatin methacrylamide hydrogel scaffolds had low density, high porosity, and interconnected macropores structure. The scaffolds appeared as an ideal support for cell survivability, proliferation, and mineralisation when seeded with adipose-derived stem cells. The hydrogel scaffolds cultured with adipose-derived stem cells were inserted into Sprague–Dawley rats subjected to a critical-size calvarial bone defect. The results of a micro-computed tomography analysis and histological staining showed new bone formation and regeneration of the defective bone, rich in osteocytes along the implantation [67].

Alginate is an anionic polysaccharide consisting of two types of copolymers, guluronic acid and mannuronic acid, that are generally found in brown algae. The high viscosity and gelling properties of the copolymers make alginate an appropriate material to be used as a scaffold for bone regeneration study, and provides greater strength and flexibility [68]. Lee et al. synthetically created an alginate injectable gel with or without collagen-binding peptide immobilisation derived from OPN, a multisubunit extracellular matrix protein. In an in vitro experiment, a culture of hBMSCs on a collagen-binding peptide-immobilised alginate gel exhibited higher cell proliferation, ALP activity, and mineral creation. The molecular mechanism underlying the osteogenic properties was the intracellular phosphorylation of Smad proteins. Moreover, the gel scaffold has also been tested in vivo by implanting the test graft into rabbits with a calvarial defect. The histological sections of the rabbit calvarial defects indicated no infection, with a good healing response, and greater new bone formation [69].

Several synthetic materials (e.g., bioglass and calcium phosphate) have been used to enhance gelatin-derived scaffolds. Kwon et al. utilised bioglass to improve the mechanical strength of methacrylated gelatin. The incorporation of these biomaterials showed promising results in bone regeneration in vivo and in vitro. The seeding of human tonsil-derived mesenchymal stem cells on bioglass-incorporated methacrylated gelatin demonstrated a non-cytotoxic cellular response and an increased bone-related gene expression of OCN, COL1, and Runx-2. Staining with Alizarin Red showed an increase in calcium content when the cells were cultured on methacrylated gelatin incorporated with bioglass. In female BALB/c mice, the bioglass embedded in methacrylated gelatin was implanted into the cranial defect. The micro-computed tomography results showed a linear increase in BV/TV with an increasing concentration of bioglass. Haematoxylin and eosin, as well as Masson’s trichome staining, indicated the enhancement in bone tissue regeneration at the defect site [70]. The study by Ye et al. incorporated bioglass into a hydrogel-derived scaffold containing gelatin and alginate. The characteristics of alginate, good biocompatibility, solubility, and viscosity, enhanced the biomechanical strength and printability of gelatin. Rat bone marrow mesenchymal stem cells that were cultured on the scaffold in the in vitro study were seen to increase cell proliferation and osteogenic differentiation directly proportional to the amount of bioglass incorporated into the scaffold [65]. Gurumurthy et al. fabricated a hydrogel scaffold by incorporating COL1 from a rat tail tendon and an elastin-like polypeptide (a genetically engineered protein polymer) with the enhancement using bioglass. The biomechanical, physical, and osteogenic properties of the scaffold were evaluated in vitro using human adipose-derived stem cells. The scaffold promoted the augmentation of live cell numbers, uniform cell spreading, cell attachment, cell proliferation, ALP activity, OCN expression, and the formation of mineralised deposits throughout the 22 days of cell culture [71].

Anada et al. synthesised hydrogel constructs, consisting of spheroids of HUVECs representing the bone marrow space, and gelatin methacrylate and octacalcium phosphate representing the cortical bone, via three-dimensional bioprinting technology. Octacalcium phosphate is a calcium phosphate that can be transformed into hydroxyapatite under physiological conditions, suggesting its role as a precursor for bones and teeth. The hydrogel constructs reinforced with octacalcium phosphate successfully induced osteogenesis and angiogenesis, evidenced by the increased proliferation of mouse multipotent mesenchymal C3H10T1/2 cells, ALP activity, and sprout formation [72]. Apart from that, hydrogel has great potential for providing localised and sustained drug delivery. A poly(ethylene glycol)-based hydrogel loaded with small interfering RNA (siRNA) and nanoparticles was generated and tested on female BALB/c mice with a mid-diaphyseal femoral fracture. Treatment with siRNA/nanoparticle-hydrogel at the fracture site allowed for the controlled release of siRNA and nanoparticles for 28 days, increased bone formation, enhanced fracture healing, and augmented biomechanical strength [73].

A chitosan-based hydrogel was developed and reinforced using metal organic frameworks (e.g., zeolitic imidazolate framework-8, ZIF-8) and modified with catechol to promote blood supply, osteogenic differentiation, bone reconstruction, and support the polymer framework of the bone graft. The properties of the synthesised hydrogel were characterised in vitro using rat bone marrow mesenchymal stem cells and in vivo using Sprague–Dawley rats with a cranial defect. The osteogenic and angiogenic activities of the rat bone marrow mesenchymal stem cells were enhanced. In animals, micro-CT analysis identified new bone formation with a thicker structure and increased BV/TV. Histological staining showed completely healed bones with mineralisation of collagen fibres, new blood vessels, and fewer inflammatory cells [74].

In brief, hydrogel exhibits superior biocompatibility, biodegradability, osteogenic capability, injectability, and drug delivery to enable optimum bone repair with minimal invasive procedures during bone graft substitution and implantation. However, hydrogel obtained solely from natural sources might be insufficient to provide greater mechanical strength, thus its application could be limited to bone regeneration purposes at non-load bearing sites. The incorporation of other synthetic materials has been proposed to strengthen the mechanical properties of hydrogel-derived scaffolds. A summary of the use of hydrogel as bone scaffolds in vivo and in vitro is presented in Table 6.

### 3.5. Polymethyl Methacrylate (PMMA)

PMMA is a synthetic resin produced from the polymerization of methyl methacrylate. It is clinically applicable in the orthopaedic field, attributable to its stable mechanical properties and surgical operability. Some disadvantages of PMMA include: (a) a high exothermic reaction causes thermal necrosis to the surrounding bone tissues; (b) it is bioinert and its poor biocompatibility inhibits cell adherence, bone formation, and causes loosening of the implantation; (c) high stiffness may increase the risk of adjacent fractures after surgery [75,76]; and (d) the toxicity of methyl methacrylate monomer released from PMMA [77,78]. To overcome these drawbacks, the addition of reinforcement agents [such as calcium phosphate, magnesium oxide (MgO), platelet gel, and linoleic acid] helps to reduce the heat produced during polymerisation and enhance osteoconductivity and osteoinductivity between the scaffold and the bone, as well as induced bone-compliant mechanical properties.

Calcium phosphate is an osteoconductive material that allows the release of calcium and phosphorus leading to the improvement of cement-to-bone bonding. The degradation of calcium phosphate subsequently induces porosities in the PMMA scaffold, thus stimulating secondary bone ingrowth. Lye et al. introduced a composite scaffold comprising β-TCP and PMMA into a rabbit model of a bilateral mandibular defect. Histological analysis indicated good scaffolding and defect bridging, no inflammation, extensive resorption of β-TCP, and the presence of intervening fibrous tissue after 15 weeks from implantation [79]. Fang et al. also developed a bio-composite bone scaffold composed of tricalcium phosphate and chitosan as additives to PMMA bone cement. However, the bio-composite exhibited a longer setting time, higher porosity after degradation, and lower compressive strength and elasticity than pure PMMA bone cement. An in vitro assay did not show any cytotoxic effect on the mouse fibroblast cell line after being seeded on the bio-composite scaffold. An in vivo experiment using eight-week-old Sprague–Dawley rats with fractures on the skull-cap demonstrated the presence of increased pore numbers between the bone and scaffold after implantation, allowing more space for bone ingrowth [80].

Metallic nanoparticles, such as magnesium oxide (MgO), possess excellent mechanical properties and durability. MgO nanoparticles release magnesium ions to activate alkaline phosphatase, enhance bone tissue formation, regulate cell functions (such as adhesion, proliferation, and migration), as well as preventing adverse reactions by the rapid degradation of magnesium. Li et al. incorporated MgO into a PMMA scaffold and tested its osteogenic capabilities on a calvarial critical bone defect in a rat model. The micro-CT and histomorphometry analysis demonstrated new bone formation and increased BMD in the animals. The better biocompatibility of the mouse embryo osteoblast precursor cells, increased formation of calcium nodules, and higher osteogenic gene expression were noted after seeding on the composite nano-metallic-polymer scaffold [81].

Platelet gel is formed by the activation of platelet-rich plasma via the addition of calcium chloride and/or thrombin. Platelet-rich plasma possesses the excellent characteristics of osteoconductivity and osteoinductivity due to its high content of growth factors which are essential for bone regeneration. A previous study demonstrated that the implantation of a PMMA scaffold added with platelet gel into a rat model of a forearm radii defect resulted in less inflammation, complete degradation of the grafting material, and increased bone formation and densities of the cartilaginous osseous tissues 8 weeks post-injury. The bone healing potential was comparable between the PMMA-platelet gel scaffold and an autograft [82]. Linoleic acid is a polyunsaturated fatty acid found in our diet, and animal or plant membranes. The addition of linoleic acid into a PMMA was found to optimise the stiffness of the PMMA, which closely resembles the mechanical properties of cancellous bone. Upon implantation into humerus and femur defects in female sheep, the normal healing process of bone tissues was observed around the implant [83].

Overall, PMMA as a single filler material for bone defects lacks biocompatibility, osteoconductivity, osteoinductivity, and osteogenic properties. In addition, the high stiffness of plain PMMA may contribute to an increased fracture risk, particularly of the adjacent osteoporotic bone. Hence, the addition of a bioactive component ensures better functionality of PMMA. A summary of the use of PMMA as bone scaffolds in vivo and in vitro is presented in Table 7.

### 3.6. Synthetic Biodegradable Polymers

The most extensively investigated synthetic biodegradable polymers for bone tissue engineering include poly(glycolic acid) (PGA), poly(lactic acid) (PLA), poly(lactic-co-glycolic acid) (PLGA), and polycaprolactone (PCL). They are linear polymers of lactic acid, glycolic acid, and/or ester synthesised through polymerisation processes.

PGA has high crystallinity with a controlled degradation rate whereby the mechanical strength decreases after two to four weeks from implantation [84]. In an in vitro experiment, a PGA scaffold allowed the proliferation and calcification of osteoblasts. The insertion of PGA scaffolds into the calvarial defects of rabbits indicated greater regenerated bone volume and mineral density [85]. PGA scaffolds loaded with collagen were implanted into a rabbit model of a calvarial bone defect. Significant fibrous connective tissue formation and a higher expression of osteogenic markers were noted after 12 weeks from treatment [86].

PLA has excellent mechanical and thermal properties. A study by Wang et al. designed pure PLA scaffolds with and without the addition of nano-hydroxyapatite. Both scaffolds were biocompatible and not toxic to rabbit bone marrow mesenchymal stem cells. The formation of calcium nodules and the expression of osteogenic bone markers were also detected in cells cultured on pure PLA and composite scaffolds, with a higher level in the composite scaffolds as compared to the pure PLA scaffold. An in vivo study demonstrated good osteointegration and biocompatibility of the scaffold, with no inflammation and no tissue necrosis after implantation of pure PLA and composite scaffolds in male New Zealand white rabbits with a femoral defect. The composite scaffold showed a higher rate of new bone growth and BV/TV than the pure PLA scaffold [87].

PLGA is a co-polymer of lactic acid and glycolic acid. The ratio between lactic acid and glycolic acid can be adjusted to control the degradation time of PLGA. Double-layered PLGA scaffolds with different pore sizes in different layers were prepared by several groups of researchers to mimic natural cartilage and subchondral bone. Their in vivo bone-repairing ability was examined in osteochondral tissue. A study by Duan et al. demonstrated that PLGA scaffolds with 100–200 μm pores in the chondral layer and 300–450 μm pores in the osseous layer exhibited the best results in stimulating the regeneration of articular cartilage and subchondral bone in a rabbit model of osteochondral defects after 12 weeks. The seeding of bone marrow mesenchymal stem cells further enhanced the osteogenic properties of the bilayer scaffolds [88,89]. Another group of investigators found that the implantation of a PLGA scaffold with 92% porosity in the cartilage layer and 77% porosity in the bone layer restored osteochondral defects at the femoral condyle of New Zealand white rabbits [90].

Reinforcement of the PLGA scaffold has been conducted by researchers. Oizumi et al. introduced octacalcium phosphate into the PLGA scaffold and its osteoinductivity was examined in rats with a femoral defect. The results showed the formation of new trabecular bone as early as 4 weeks and the bone defect was almost completely bridged 8 weeks after the octacalcium phosphate/PLGA scaffold implantation, and its bone repair ability was superior when compared to PLGA alone. The results from micro-computed tomography supported the histological findings, indicating higher BV/TV, Tb.Th, and lower Tb.Sp eight weeks post-implantation. Accumulation of tartrate-resistant acid phosphatase-positive osteoclast-like cells was observed at week 4, whereas osteocalcin-positive osteoblastic cells appeared at week 8, reiterating the biodegradation of the implanted scaffold followed by new bone formation at the defect site [91].

Chen et al. incorporated icaritin and tricalcium phosphate into the PLGA scaffold. Icaritin serves as an exogenous growth factor, whereas tricalcium phosphate balances the pH during the degradation of PLGA to reduce inflammation. The composite scaffold resulted in the formation of mineralised bones and blood vessels when implanted in an ulnar-bone-defect rabbit model [92]. In another study, Lai et al. enhanced the PLGA scaffold with β-TCP and magnesium, which was then tested in a steroid-associated-osteonecrosis rabbit model. Magnesium appears as an ideal enhancer due to its mechanical properties and elastic modulus which mimics the natural cortical bone. The implantation of a PLGA/β-TCP/magnesium scaffold enhanced new blood vessel and bone formation, improved bone microstructure and strength, as well as increased osteogenic and angiogenic factors in the rabbits. The scaffold was proven to be safe, without an immune response, as well as liver and kidney function parameters were at the physiological level [93].

PCL is a biodegradable polyester with thermal stability, elasticity, flexibility, permeability to small drug molecules, and biocompatibility, but a slow degradation rate when compared to other synthetic biodegradable polymers. With these features, it has been suggested that PCL could be more appropriate as a long-term implant or used in combination with other polymer biomaterials [84]. Yao et al. incorporated PLA into PCL to overcome the low elasticity and flexibility of PLA, as well as the slow degradation rate and decreased cell surface adhesion of PCL. An in vitro study pointed out the enhancement of cell viability and osteogenic differentiation in human mesenchymal stem cells cultured on the fabricated scaffolds. The in vivo study found that new bone formation was observed in the male C57BL/6NHsd mice with a critical-sized cranial bone defect implanted with the composite scaffold [94]. The blending of PCL and PLA has been proven to increase BMD at the defect site of distal ulna in mature, healthy New Zealand rabbits [95]. Hassanajili et al. developed a composite scaffold by combining PCL, PLA, and hydroxyapatite. The composite scaffold allowed cell adhesion, viability, proliferation, and mineral deposition in MG63 cells [96].

**Figure 2:**
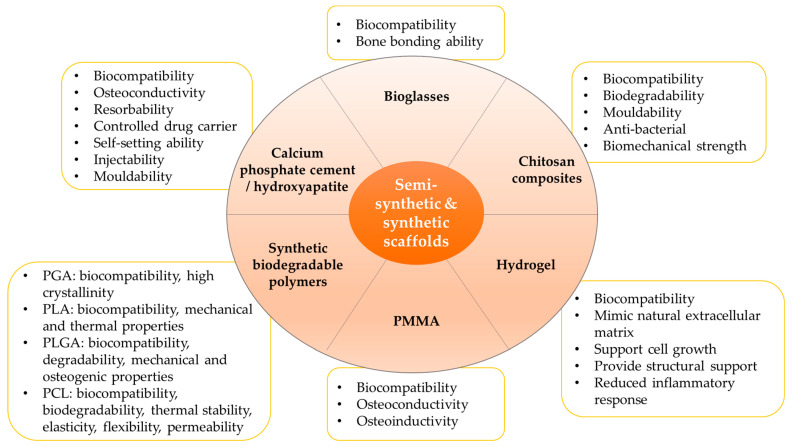
The properties of semi-synthetic and synthetic scaffolds in bone tissue engineering. Abbreviations: PCL, polycaprolactone; PGA, poly(glycolic acid); PLA, poly(lactic acid); PLGA, poly(lactic-co-glycolic acid).

In general, synthetic biodegradable polymers offer several advantages. First, the degradation products (such as hydroxyacetic acid, lactic acid, and glycolic acid) can be safely metabolised and excreted through physiological metabolic cycles. Second, they have a high tensile and elastic modulus, but their strength decreases with the gradual degradation of the implant time. This dynamic characteristic provides good mechanical properties to support the bone defect site. Third, the polymers can be incorporated with various bioactive factors to facilitate bone regeneration [97]. The excellent biocompatibility, controlled degradation, suitable mechanical strength, and ability to act as a sustained delivery system to slowly release bioactive factors suggest the application of synthetic biodegradable polymers as load-bearing fillers in bone repair and regeneration. However, the degradation products of these polymers can be acidic, which may further accelerate the implant’s degradation rate and induce inflammatory reactions. The inclusion of other biomaterials can be considered to ensure an optimal balance in pH. A summary of the use of synthetic biodegradable polymers as bone scaffold in vivo and in vitro is presented in Table 8. The properties of semi-synthetic and synthetic scaffolds in bone tissue engineering have been summarised in Figure 2.

## 4. Perspectives

The naturally derived scaffolds better mimic the native architecture of a bone than synthetic scaffolds in allowing and supporting cellular growth [98]. Mebarki et al. reported a higher accessible surface area for cell invasion and better cell adhesion of hBMSCs on Tutoplast^®^-processed human bone as compared to hydroxyapatite/β-TCP scaffold, due to the open pore structure and interconnectivity of the Tutoplast^®^-processed human bone. A higher receptor activator of the nuclear factor-kappa B ligand (RANKL) expression was noted when hBMSCs were loaded on the hydroxyapatite/β-TCP scaffold relative to the Tutoplast^®^-processed human bone. In vivo, the frequency of bone formation was higher in mice implanted with the natural Tutoplast^®^-processed human bone as compared to those implanted with the synthetic hydroxyapatite/β-TCP scaffold [27]. Although both the natural and synthetic scaffolds showed great osteoconduction, osteoinduction, and osteogenetic properties, this study reiterated that the natural endogenous bone structure possesses a large surface area and spongy architecture favouring higher cell-seeding efficiency as compared to the single block structure of hydroxyapatite/β-TCP. Furthermore, natural-derived scaffolds from animals or plants are often easy to obtain as they are freely available. However, the natural-derived scaffolds require decellularisation that may disrupt the extracellular matrix. The preservation of the extracellular matrix is essential for favourable cell attachment, migration, and differentiation, suitable mechanical performance, as well as not eliciting an immune-mediated rejection [99]. Demineralisation of the bone matrix may compromise the support of physical loads, thus hindering its application at weight-bearing sites and affecting the pore size, making them difficult for cell penetration.

**Figure 3:**
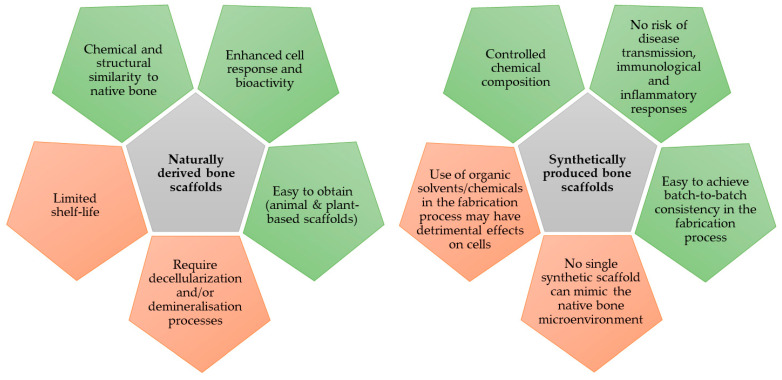
The merits (green boxes) and caveats (orange boxes) of natural and synthetic scaffold in bone tissue engineering.

Synthetic scaffolds offer several advantages over natural scaffolds. Firstly, the chemical composition is controllable to ensure excellent mechanical strength. For instance, the combination of hydroxyapatite with silica mixture provides a greater mechanical strength that supports tensile stress and manages to bear loads [100]. Secondly, the use of scaffolds from bioengineered materials carry a low risk of disease transmission, as well as negligible immunological and inflammatory responses. Thirdly, the fabrication of synthetic scaffolds can be conducted on a large scale and easily achieve high batch-to-batch consistency even at different time points. The characteristics of naturally derived bone scaffolds harvested from various parts of the bone can differ from each other. On the other hand, synthetic materials are often used together in combination because a single synthetic material on its own has reduced biosimilarity to native bone. For example, hydrogel has lower biomechanical strength, while bioglass lacks collagen which is not favourable for bone cell growth. Hence, one or more substances may be required for fabricating a composite scaffold (Figure 3).

Limitations to the current evidence need to be addressed. Limited studies have been conducted to compare the potential use of natural and synthetic scaffolds under the same experimental setting. Moreover, although the osteogenic properties of natural and synthetic scaffolds have been proven, their vascularisation capacity should not be neglected. More comprehensive research is warranted to improve vascularisation by allowing cells to form microvasculatures in the scaffold prior to implantation. Several considerations in developing bone scaffolds for bone tissue engineering need to be acknowledged. The biocompatibility of a bone scaffold refers to the ability of the material to function in a living organism with no risk of immunological rejection and inflammatory response. This remains the indispensable feature an ideal material for application in bone tissue engineering must fulfil. Synthesising an engineered bone that mimics an in vivo situation requires the optimisation of the porosity–strength relationship. Adequate mechanical strength is needed from the time of implantation until the bone remodelling process is complete. Meanwhile, an ideal porosity with a large surface area is essential for osteointegration between the scaffold and host tissues. Another aspect that limits the clinical application of bone scaffolds is the healing process, which could vary depending on the patient’s condition. A commercially viable bone scaffold should be cost-effective and able to be scaled up into batch production with good manufacturing practices and a long shelf availability to be translated into a clinical application. However, the bone repair process is rapid in young and healthy individuals, but slows down in individuals who are elderly and who have medical conditions. Hence, a tailor-made bone graft is a way forward which could lead to a higher cost of fabrication.

## 5. Conclusions

Both natural and synthetic scaffolds have their unique advantages and disadvantages, whereby the former is ideal for its bioactivity and biocompatibility, whereas the latter enables better control of mechanics, geometry, porosity, and degradation rate. The current advancements in bone tissue engineering should focus on the development of bone substitutes with a combination of the three main aspects, including scaffold, cells, and osteoconductive stimuli, to better mimic the physiological condition of endogenous bone. The combination of natural and synthetic materials could be a better choice given the merits of the intrinsic biocompatibility of natural scaffolds and the physicochemical properties of synthetic materials in the fabrication of biocomposites in bone tissue engineering.

## Figures and Tables

**Table 2:** The use of plant-derived scaffold in vivo and in vitro for bone regeneration.

Type of Scaffold	Type of Model	Findings	Reference
Apple-, broccoli-, sweet pepper-, carrot-, persimmon-, and jujube-derived scaffold	Pluripotent stem cells	-Increased cell viability in apple-derived scaffold. -Cells remained poorly spread and proliferated in other types of plant scaffolds.	[34]
Apple-derived scaffold	Pluripotent stem cells undergoing osteoblastic differentiation	-Increased cell viability, proliferation, mineralisation, expression of OCN, COL1, and sclerostin (SOST).	
	Male Sprague–Dawley with calvarial defect	-Increased bone volume/total volume (BV/TV), bone regeneration area, calcium deposition, and blood vessel formation with no sign of inflammation.	
Carrot-derived scaffold	MC3T3-E1 cells	-Readily available, low-cost, and ethical compared to animal-derived scaffolds. -Supported the adhesion, proliferation and osteogenic differentiation of MC3T3-E1 pre-osteoblasts. -Increased ALP activity and the presence of bone sialoprotein.	[36]

Abbreviations: ALP, alkaline phosphatase; BMD, bone mineral density; BV/TV, bone volume/total volume; COL1, collagen I; hBMSCs, human bone marrow stromal cells; MC3T3-E1, mouse osteoblast-like cells; OCN, osteocalcin; SOST; sclerostin.

**Table 3:** The use of calcium phosphate cement and hydroxyapatite scaffold in vivo, in vitro, and in humans for the evaluation of bone regeneration.

Type of Scaffold	Type of Enhancers	Type of Model	Findings	Reference
β-TCP	-	Immunodeficient CD-1 nude mice	-Increased total bone formation, mature bone formation, and neovascularisation.	[38]
Hydroxyapatite/β-TCP	-	Male SCID mice	-Bone formation and cell adhesion on the scaffold.	[27]
β-TCP/CS	-	Male New Zealand white rabbits with circular bicortical critical-size cranial defect	-Decreased Gr.V/TV and Gr.Ar. -Increased BV/TV, OV/TV, B.Ar, O.Ar, O.Pm, Oc.N, and mineralisation marginally. -No change in Ob.N, Ob.Pm, and Fb.Ar.	[39]
Calcium phosphate cement	-	Patients with trochanteric fracture (*n* = 21; aged 66–95 years)	-Improved fracture stability.	[40]
Calcium phosphate cement	-	Patients with acute distal radial fracture (*n* = 48; aged >65 years)	-No association for flexion arc, extension arc, supination arc, pronation arc, grip strength, VAS scores, MMWS, DASH scores, or radiographic parameters. -No association in mean volumes of metaphyseal defects.	[41]
Hydroxyapatite and calcium phosphate cement	Calcium silicate	Goat bone marrow-derived mesenchymal stem cells	-Increased viability of cells, cell adhesion, cell migration, ALP activity, osteopontin (OPN), and OCN in silica-coated hydroxyapatite compared to pure hydroxyapatite.	[43]
Hydroxyapatite	Dopamine-modified alginate and quaternised chitosan	Human chondrocytes and fibroblasts	-Gradient scaffold promotes new bone formation and accelerates bone defect repair in vivo compared to the homogenous scaffold.	[44]
β-TCP	Poly(1,8-octanediol-co-citrate) and cerium oxide nanoparticles	Sprague–Dawley rats	-Scaffold degradation.	[45]
		Primary human osteoblasts	-No cytotoxicity, good cell attachment, proliferation, and mineralisation on the scaffolds.	

Abbreviations: ALP, alkaline phosphatase; B.Ar, bone area; BV/TV, bone volume/total volume; β-TCP, β-tricalcium phosphate; DASH, disabilities of the arm, shoulder, and hand; Fb.Ar, fibrous connective tissue area; Gr.Ar, graft area; Gr.V/TV, graft volume/tissue volume; MMWS, modified Mayo wrist scores; O.Ar, osteoid area; Ob.N, osteoblast number; Ob.Pm, osteoblast perimeter; OCN, osteocalcin; Oc.N, osteoclast number; O.Pm, osteoid perimeter; OV/TV, osteoid volume/tissue volume; SCID, severe combined immunodeficient; VAS, visual analog scale.

**Table 4:** The use of bioglass as bone scaffold in vitro and in vivo for the evaluation of bone regeneration.

Type of Scaffold	Type of Enhancers	Type of Model	Findings	Reference
Bioglass	-	Primary human osteoblast and HUVECs	-Scaffolds were non-toxic to cells and showed increased cell proliferation.	[47]
Bioglass	-	Male Wistar rats with non-critical-sized tibial defect	-Complete degradation, no inflammation, new bone formation, and increased mechanical strength.	[48]
Bioglass	-	Male Wistar Lineage rats with femoral bone defect	-Lower BV/TV and Tb.N were found in the animals receiving a bioglass scaffold than those receiving autogenous bone. -No difference in Tb.Th or Tb.Sp were found between the groups receiving a bioglass scaffold and autogenous bone.	[49]
	Niobium		-No difference in BV/TV, Conn.D, Tb.Th, or Tb.N between the groups receiving a niobium-supplemented bioglass scaffold and autogenous bone.	
Bioglass	-	Calvaria bone defect rat model	-BV/TV, Tb.Th, Tb.Sp, and Tb.N were comparable between the animals implanted with a bioglass nanoceramic composite, with and without raloxifene.	[50]
	Raloxifene			
Bioglass	Boron	Rat bone marrow mesenchymal stem cells	-Scaffolds were non-toxic to cells and showed proper cell attachment.	[51]
		Male New Zealand white rabbit with mandibular defect	-Fast bone repair, scaffold degradation, bone regeneration with increased BMD, BV/TV, Tb.Th, and decreased Tb.Sp.	
Baghdadite	Bioglass	Merino wethers with critical-sized segmented bone defect at right tibia	-No difference in torsional stiffness, ultimate torsional strength, bone volume, bone bridging, or area of infiltrating bone between baghdadite scaffolds, with and without bioglass nanoparticles.	[52]

Abbreviations: BMD, bone mineral density; BV/TV, bone volume/total volume; Conn.D, connectivity density; HUVECs, human umbilical vein endothelial cells; Tb.Th, trabecular thickness; Tb.N, trabecular number; Tb.Sp, trabecular separation.

**Table 5:** The use of chitosan-composites as bone scaffolds in vitro and in vivo for the evaluation of bone regeneration.

Type of Scaffold	Type of Enhancers	Type of Model	Findings	Reference
Chitosan	Hydroxyapatite	MC3T3-E1 cells	-Increased cell number, osterix expression, ALP activity, and mineralisation.	[58]
		Male C57BL/6J mice with rod-fixated tibia fracture	-Increased total bone volume, tissue volume, Tb.N, Conn.D, polar moment of inertia, and osteoid volume. -Decreased Tb.Sp and trabecular pattern factor. -Presence of cartilage matrix	
Chitosan	Hydroxyapatite	MC3T3-E1 cells	-Increased ALP, cell proliferation, and cell viability.	[57]
Chitosan	Nano-sized silica	Mice bone marrow stromal cells	-Non-cytotoxic.	[59]
Chitosan	Gelatin and nano-silica	MG63 cells	-Non-cytotoxic, proper cell attachment, and increased cell proliferation.	[60]
Chitosan	Two dimensional-layered nanoparticles consisting of Mg-Al-PO_4_-layer double hydroxide and nanoclay	NIH 3T3 cells	-Enhanced mechanical property as compared to pure chitosan. -Increased cell viability and cell proliferation within pores.	[61]
		Albino Wistar rats with femoral bone defect	-Faster bone healing, denser bone morphology, and higher osteoblast number as compared to pure chitosan. -No changes in the levels of liver enzymes (aspartate aminotransferase and alanine aminotransferase), urea, or creatinine.	
Chitosan	Polytrimethylene carbonate/polylactic acid (PLLA)/oleic acid-modified hydroxyapatite/vancomycin hydrochloride	MC3T3-E1 cells	-Slow biodegradability. -Increased osteogenic proliferation, adhesion of osteoblasts, and high mechanical and surface strength.	[62]

Abbreviations: ALP, alkaline phosphatase; Conn.D, connectivity density; MC3T3-E1, mouse osteoblast-like cells; Mg-Al-PO4, magnesium aluminium phosphate oxide; MG63, human osteoblast cells; NIH 3T3, mouse embryonic fibroblasts; Tb.N, trabecular number; Tb.Sp, trabecular separation.

**Table 6:** The use of hydrogel as bone scaffold in vitro and in vivo for the evaluation of bone regeneration.

Type of Scaffold	Type of Enhancers	Type of Model	Findings	Reference
Gelatin methacryloyl hydrogel	-	4-month-old New Zealand white rabbit with large segmental defect at the radius	-Implantation of hydrogel increased new bone formation, healing rate, bone remodelling, and bone volume. -Higher degradation rate, formation of fibrous tissue between trabeculae, cell number, and bone marrow tissue were observed in animals implanted with hydrogel than control.	[66]
		Bone marrow mesenchymal stem cells (BMSCs)	-Increased cell proliferation, chemotactic effect, regulation of COL1, ALP, OCN, Runx-2, OPN, and bone mineralisation.	
Gelatin methacylamide hydrogel	-	ADSCs	-Increased cell proliferation, ALP activity, and mineralisation.	[67]
		Sprague–Dawley rats with critical-size calvarial bone defect	-Increased regeneration of defective bone.	
CBM peptide-alginate gel	-	Rabbits with calvarial defect	-Increased new bone formation and BMD.	[69]
		hBMSCs	-Increased ALP, Smad1/5/8, cell proliferation, osteogenesis, calcein uptake, and BMD.	
Methacrylated gelatin hydrogel	Bioglass	Human tonsil-derived mesenchymal stem cells	-Increased mechanical strength and increased osteogenic differentiation of cells.	[70]
		Female balb-C mice with cranial defect	-Increased bone formation in the animal.	
Gelatin-alginate hydrogel	Bioglass	Rat bone marrow mesenchymal stem cell	-Proliferation of viable cells. -Increased osteogenic differentiation.	[65]
COL1 and elastin-like polypeptide-based hydrogel	Bioglass	Human adipose-derived stem cells	-Increased cell viability, spreading, attachment, proliferation, ALP activity, OCN content, and mineralisation.	[71]
Gelatin methacrylate hydrogel	Octacalcium phosphate	Mouse multipotent mesenchymal C3H10T1/2 cells	-Increased cell proliferation, cell differentiation, and angiogenesis.	[72]
Poly(ethylene glycol)-based hydrogel	siRNA/nanoparticle	Female BALB/c mice with mid-diaphyseal femoral fracture	-Increased callus area, cartilage formation, and bone area. -Decreased unmineralised cartilage and fibrotic tissue formation.	[73]
Chitosan-based hydrogel	Zeolitic imidazolate framework-8	Rat bone marrow mesenchymal stem cells	-Increased ALP, COL1, OCN, and vascular endothelial growth factor (VEGF). -Extracellular matrix mineralisation and has antibacterial effects.	[74]
		Male Sprague–Dawley (8 weeks) cranial defect model	-Increased BV/TV, BMD, ALP, OCN, and mineralisation. -Increased angiogenesis. -Decreased inflammatory cells.	

Abbreviations: ADSCs, adipose derived stem cells; ALP, alkaline phosphatase; BMD, bone mineral density; BV/TV, bone volume/total volume; COL1, collagen type 1; OCN, osteocalcin; OPN, osteopontin; Runx-2, Runt-related transcription factor 2; siRNA, small interfering ribonucleic acid.

**Table 7:** The use of PMMA as bone scaffold in vitro and in vivo for the evaluation of bone regeneration.

Type of Scaffold	Type of Enhancers	Type of Model	Findings	Reference
PMMA	Tricalcium phosphate and chitosan	Mouse fibroblastic cell line	-Non-cytotoxic.	[80]
		Sprague–Dawley rats with skull-cap fractures	-Increased bone ingrowth.	
PMMA	β-TCP	New Zealand white rabbits with bilateral mandibular defect	-Bone ingrowth, no inflammation, and thin fibrous tissue observed at the surface of bone-cement.	[79]
PMMA	MgO nanoparticles	Mouse embryo osteoblast precursor cells	-Better biocompatibility, presence of calcium nodules, and higher osteogenic gene expression.	[81]
		Sprague–Dawley rats with calvarial critical bone defect	-New bone formation and higher BMD.	
PMMA	Platelet gel	Sprague–Dawley rats with forearm radii defect	-Less inflammation and increased density of cartilage and osseous tissue.	[82]
PMMA	Linoleic acid	Female sheep with humerus and femur defects	-Good biocompatibility and harmless tissue healing, but lacks mechanical strength.	[83]

Abbreviations: BMD, bone mineral density; β-TCP, β-tricalcium phosphate; MgO, magnesium oxide; PMMA, polymethyl methacrylate.

**Table 8:** The use of synthetic biodegradable polymers as bone scaffold in vitro and in vivo for the evaluation of bone regeneration.

Type of Scaffold	Type of Enhancers	Type of Model	Findings	Reference
PGA	-	MC3T3-E1 cells	-Increased cell proliferation, differentiation, and calcification.	[85]
		Rabbits with calvarial bone defect	-Increased bone mineral density and connective tissue formation.	
PGA	Collagen	New Zealand white rabbits with calvarial bone defect	-Formation of fibrous connective tissue and bony bridging at the defect site. -Damage area at the defect site was reduced. -Increased ALP and sialoprotein expressions.	[86]
PLA	Nano-hydroxyapatite	Rabbit bone marrow mesenchymal stem cells	-Formation of calcium nodules. -Expression of Runx-2, COL1, OPN, and bone morphogenetic protein-2 (BMP-2).	[87]
		Male New Zealand white rabbit femoral defect model	-Good osteointegration and biocompatibility. -No inflammation and no tissue necrosis. -New bone growth. -Composite scaffolds have higher bone growth and BV/TV than PLA scaffolds.	
PLGA	-	New Zealand white rabbit with osteochondral defect at femoral condyles	-Increased compressive modulus. -No inflammation at the synovial membrane and joint tissues. -Increased percentage of hyaline cartilage. -Increased aggrecan, COL1, and COL2 levels.	[88,89,90]
PLGA	Octacalcium phosphate	Male Sprague–Dawley rats with femoral defect	-Increased BV/TV and Tb.Th, and decreased Tb.Sp. -Accumulation of osteoclast-like cells at week 4 and osteocalcin-positive osteoblast cells at week 8.	[91]
PLGA	Tricalcium phosphate, icaritin	Male New Zealand white rabbit with ulnar-bone-defect model	-Neovascularisation and new mineralised bone formation.	[92]
PLGA	β-TCP and magnesium	Male New Zealand white rabbit with osteonecrosis induced by methylprednisolone	-Increased BV/TV, BMD, Tb.N, bone surface/tissue surface, and decreased Tb.Sp. -Increased energy and maximum load of bone. -Increased expression of BMP-2 and vascular endothelial cell growth factor A.	[93]
PCL and PLA	-	Human mesenchymal stem cell	-Enhance cell viability and osteogenic differentiation.	[94]
		Male C57BL/6NHsd mice critical-sized cranial bone defect	-New bone formation.	
PCL and PLA	-	New Zealand rabbit with distal ulna defect	-Great bone regeneration. -Higher BMD than pure PLA.	[95]
PCL and PLA	Hydroxyapatite	MG63 cells	-Cell adhesion, cell viability and mineral deposition.	[96]

Abbreviations: β-TCP, β-tricalcium phosphate; BMD, bone mineral density; BMP-2, bone morphogenetic protein-2; BV/TV, bone volume/total volume; COL1, collagen type 1; COL2, collagen type 2; MC3T3-E1, mouse osteoblast-like cells; OPN, osteopontin; PCL, polycaprolactone; PGA, poly(glycolic acid); PLA, poly(lactic acid); PLGA, poly(lactic-co-glycolic acid); Runx-2, Runt-related transcription factor 2; Tb.N, trabecular number; Tb.Sp, trabecular separation; Tb.Th, trabecular thickness.

## Data Availability

Not applicable.
